# Using a collaborative learning health system approach to improve disease activity outcomes in children with juvenile idiopathic arthritis in the Pediatric Rheumatology Care and Outcomes Improvement Network

**DOI:** 10.3389/fped.2024.1434074

**Published:** 2024-08-02

**Authors:** Julia G. Harris, Catherine A. Bingham, Sheetal S. Vora, Cagri Yildirim-Toruner, Michelle Batthish, Danielle R. Bullock, Jon M. Burnham, Danielle C. Fair, Kerry Ferraro, Suhas Ganguli, Mileka Gilbert, Beth S. Gottlieb, Olha Halyabar, Melissa M. Hazen, Ronald M. Laxer, Tzielan C. Lee, Alice Liu, Daniel J. Lovell, Melissa L. Mannion, Edward J. Oberle, Nancy Pan, Michael Shishov, Jennifer E. Weiss, Esi M. Morgan

**Affiliations:** ^1^ Department of Pediatrics, Children’s Mercy Kansas City and University of Missouri-Kansas City School of Medicine, Kansas City, MO, United States; ^2^ Department of Pediatrics, Penn State Children’s Hospital and Penn State College of Medicine, Hershey, PA, United States; ^3^ Department of Pediatrics, Atrium Health Levine Children’s Hospital and Wake Forest School of Medicine, Charlotte, NC, United States; ^4^ Department of Pediatrics, Texas Children’s Hospital and Baylor College of Medicine, Houston, TX, United States; ^5^ Department of Pediatrics, McMaster Children’s Hospital and McMaster University, Hamilton, ON, Canada; ^6^ Department of Pediatrics, University of Minnesota and M Health Fairview Masonic Children’s Hospital, Minneapolis, MN, United States; ^7^ Department of Pediatrics, Children’s Hospital of Philadelphia, Philadelphia, PA, United States; ^8^ Department of Pediatrics, Medical College of Wisconsin and Children’s Wisconsin, Milwaukee, WI, United States; ^9^ Department of Pediatrics, Hackensack University Medical Center and Hackensack Meridian Health, Hackensack, NJ, United States; ^10^ Department of Pediatrics, Medical University of South Carolina, Charleston, SC, United States; ^11^ Department of Pediatrics, Cohen Children’s Medical Center and Northwell, New Hyde Park, NY, United States; ^12^ Department of Pediatrics, Boston Children’s Hospital, Boston, MA, United States; ^13^ Departments of Pediatrics and Medicine, The Hospital for Sick Children, St. Michael’s Hospital, and the University of Toronto, Toronto, ON, Canada; ^14^ Department of Pediatrics, Stanford Medicine Children’s Health and Stanford University, Stanford, CA, United States; ^15^ Seattle Children’s Research Institute, Seattle, WA, United States; ^16^ Department of Pediatrics, Cincinnati Children’s Hospital Medical Center, Cincinnati, OH, United States; ^17^ Department of Pediatrics, University of Alabama at Birmingham, Birmingham, AL, United States; ^18^ Department of Pediatrics, Nationwide Children’s Hospital and The Ohio State University, Columbus, OH, United States; ^19^ Department of Pediatrics, Hospital for Special Surgery and Weill Medical College of Cornell University, New York, NY, United States; ^20^ Department of Pediatrics, Phoenix Children’s Hospital, Phoenix, AZ, United States; ^21^ Department of Pediatrics, Seattle Children’s Hospital & University of Washington School of Medicine, Seattle, WA, United States

**Keywords:** juvenile arthritis, quality improvement, outcome measures, pediatrics, rheumatology, registries, collaborative learning

## Abstract

**Introduction:**

The Pediatric Rheumatology Care and Outcomes Improvement Network (PR-COIN) is a North American learning health network focused on improving outcomes of children with juvenile idiopathic arthritis (JIA). JIA is a chronic autoimmune disease that can lead to morbidity related to persistent joint and ocular inflammation. PR-COIN has a shared patient registry that tracks twenty quality measures including ten outcome measures of which six are related to disease activity. The network's global aim, set in 2021, was to increase the percent of patients with oligoarticular or polyarticular JIA that had an inactive or low disease activity state from 76% to 80% by the end of 2023.

**Methods:**

Twenty-three hospitals participate in PR-COIN, with over 7,200 active patients with JIA. The disease activity outcome measures include active joint count, physician global assessment of disease activity, and measures related to validated composite disease activity scoring systems including inactive or low disease activity by the 10-joint clinical Juvenile Arthritis Disease Activity Score (cJADAS10), inactive or low disease activity by cJADAS10 at 6 months post-diagnosis, mean cJADAS10 score, and the American College of Rheumatology (ACR) provisional criteria for clinical inactive disease. Data is collated to measure network performance, which is displayed on run and control charts. Network-wide interventions have included pre-visit planning, shared decision making, self-management support, population health management, and utilizing a Treat to Target approach to care.

**Results:**

Five outcome measures related to disease activity have demonstrated significant improvement over time. The percent of patients with inactive or low disease activity by cJADAS10 surpassed our goal with current network performance at 81%. Clinical inactive disease by ACR provisional criteria improved from 46% to 60%. The mean cJADAS10 score decreased from 4.3 to 2.6, and the mean active joint count declined from 1.5 to 0.7. Mean physician global assessment of disease activity significantly improved from 1 to 0.6.

**Conclusions:**

PR-COIN has shown significant improvement in disease activity metrics for patients with JIA. The network will continue to work on both site-specific and collaborative efforts to improve outcomes for children with JIA with attention to health equity, severity adjustment, and data quality.

## Introduction

Pediatric Rheumatology Care and Outcomes Improvement Network (PR-COIN) is a learning health network (LHN) designed to improve and advance the care of children with juvenile idiopathic arthritis (JIA) (1, 2). JIA is a chronic autoimmune disease affecting about 1 in 1,000 children that can lead to life-long damage to joints from arthritis and vision loss from uveitis without proper care. LHNs leverage multisite stakeholders including patients, families, medical providers, other healthcare staff, researchers, and community organizations working together with a common goal and sense of urgency to develop knowledge from data to deliver better clinical care and improve health outcomes more equitably.

Using methodology from the Model for Improvement, the Institute for Healthcare Improvement Breakthrough Series, and with quality improvement (QI) guidance and initial coordination from the James Anderson Center for Health Systems Excellence at Cincinnati Children's Hospital Medical Center, PR-COIN modeled its beginnings after ImproveCareNow, a pediatric inflammatory bowel disease multi-center collaborative of currently over 100 participating medical centers for its significant achievements including sustained remission rates in their patient population (3–5). Eager to achieve similar improvements in JIA outcomes, PR-COIN launched in 2011 as an improvement collaborative with an inaugural membership of 12 centers and started its journey to achieve extraordinary rates of disease control in JIA while using clinical data for QI and research with a goal to accurately and reliably measure and report performance on process and outcome quality measures to drive improved outcomes (1).

JIA is a lifelong disease with a high risk of morbidity related to both the disease and its treatments, potentially causing permanent damage to joints and eyes. Early diagnosis and timely, effective treatment are crucial as JIA can significantly impact a child's growth, development, and quality of life (6). A 17-year follow-up study of patients with JIA revealed a generally favorable outcome for most patients, yet ocular involvement remained prevalent (7). Despite good physical and social functioning, many patients expressed feeling burdened by their condition, with current disease activity strongly influencing functional status. Predictors of long-term active disease include early onset, specific joint involvement, and elevated inflammatory markers (8).

Over the past two decades, several outcome measures have been developed and validated to monitor how JIA progresses and to help manage it effectively at the point of care. These measures are designed to provide a comprehensive view of a patient's condition, that allow for tailoring treatments to individual needs and monitoring overall disease progression and response to therapy. Key measures focus on clinical disease activity, functional status, radiographic outcomes, laboratory markers, and patient-reported outcomes.

Utilization of outcome measures is essential because it enables providers to better track disease progression, assess treatment efficacy, effectively monitor disease progression, and implement timely interventions for better outcomes. PR-COIN utilizes QI methodologies to enhance collection and monitoring of outcome measures in JIA. By systematically analyzing and improving the care processes, PR-COIN aims to enhance the effectiveness and efficiency of JIA management.

PR-COIN employs various QI strategies, such as Plan-Do-Study-Act cycles, to iteratively test and refine changes in clinical practice. Through collaborative efforts among healthcare providers, researchers, and patients, PR-COIN identifies areas for improvement in the utilization of outcome measures in clinical care, such as enhancing the sensitivity of detection, standardizing assessment methods, and integrating patient-reported outcomes. By incorporating feedback from stakeholders and continuously evaluating the impact of interventions, PR-COIN ensures that improvements in outcome measures are evidence-based and patient-centered.

Moreover, PR-COIN leverages data-driven approaches to monitor progress and benchmark performance across different healthcare settings. By collecting and analyzing real-world data on JIA outcomes, PR-COIN identifies best practices and facilitates knowledge sharing among participating institutions. This collaborative learning environment accelerates the dissemination of effective strategies for enhancing outcome measures in JIA care.

PR-COIN has a shared patient registry that currently tracks 20 quality measures (1). Quality measure categories include outcome, process, balancing, and data quality measures. PR-COIN has ten quality measures measuring health care outcomes including six related to disease activity and four patient-reported outcomes. The focus of this manuscript is reporting of the disease activity outcome measures. The PR-COIN collaborative's global aim in 2021 was to increase the percent of patients with oligoarticular or polyarticular JIA in an inactive or low disease activity state from 76% to 80% by the end of 2023.

## Materials and methods

This manuscript utilized the SQUIRE 2.0 reporting guidelines (9).

### Context

PR-COIN uses a collaborative learning health system approach to improve quality of care and outcomes for children with JIA (10–12). PR-COIN currently has 23 participating sites from academic pediatric medical centers throughout the United States and Canada. PR-COIN is led by a coordinating center which provides quality improvement consultation, quality improvement education, maintenance of certification opportunities, data management, data analytics, legal and regulatory supervision, project development and oversight, and overall support to the network. Additionally, PR-COIN has seven operating committees directing Measures, Outcomes, Informatics, Scientific Development and Oversight (Research), Engagement, Finance and External Partnerships, and Education activities all led by volunteer members. The leaders for each committee together form the Executive Committee along with the principal investigator to prioritize network-wide initiatives in line with the stated mission and vision of PR-COIN (2). Elected members join the committee leads to form the Steering Committee to provide additional representative network oversight.

PR-COIN member centers have local QI teams that vary in composition by site, but typically consist of a physician champion, other providers including rheumatologists, pediatric learners (e.g., rheumatology fellow, pediatric resident, medical student), occupational and physical therapists, nurses, and other staff including medical assistants, social workers, administrative staff, and research staff. Some centers receive local QI improvement specialist support from their institution. Most valuable is the personal contribution of patients and families to PR-COIN QI work at both the local team and network committee level lending their experience and expertise. Patients and families contribute to workgroups of specific interests, educational presentations, development of QI tools and other items dealing with specific challenges unique to the JIA population. Local team members conduct QI projects of greatest value to their site using the Model for Improvement and rapid plan-do-study-act cycles, contribute to network led initiatives, and “share seamlessly and steal shamelessly” the best practices presented at monthly action-period calls and twice-yearly learning sessions held in person and virtually to accommodate participation from all members.

Data from PR-COIN sites are collected at the point-of-care, with the goal to collect data on every patient at every visit, in order to calculate performance on JIA quality measures. PR-COIN has a shared registry platform, operated by vendor Hive Networks, allowing individual sites to see both site and aggregate data in a centralized platform to monitor quality measure performance (1, 13)*.* The PR-COIN registry contains data from over 13,500 registered patients, over 7,200 of whom are active patients, and greater than 89,000 patient visits.

### Interventions

#### QI tools

**Figure 1:**
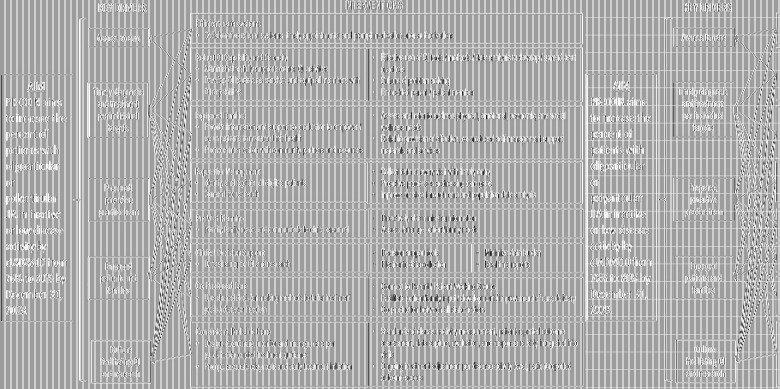
Key driver diagram highlighting our aim, primary drivers, and potential interventions.

The collaborative utilized QI tools in their improvement efforts including creation of a key driver diagram (Figure 1) to identify drivers and interventions to help achieve their aims. The collaborative and many sites also utilized other QI tools including process maps, cause and effect diagrams, failure modes and effects analyses, and pareto charts. PR-COIN sites have conducted numerous interventions to help improve performance on quality measures, including disease activity outcome measures. Interventions have been both site-specific and network-wide. Some interventions or interventional themes have spanned multiple sites as PR-COIN has facilitated several network-wide initiatives.

PR-COIN uses strategies advocated by the Chronic Care Model (CCM) (14). The CCM is an organizational approach to delivery of healthcare for chronic diseases and includes six key domains in which high quality health care can be developed through QI efforts including the community, the health system, self-management support, delivery system design, clinical decision support, and clinical information systems (14). Studies suggest employment of the CCM improves healthcare delivery and outcomes for patients with chronic diseases (15, 16).

#### Pre-visit planning

One early network intervention adopted by PR-COIN was the use of pre-visit planning (PVP) (17). PVP is the process whereby the clinical team reviews the electronic health record (EHR) and may also survey patients to make sure that the data that are needed for the clinic visit is readily available at point of care (18). When health care teams are prepared for clinic visits, valuable patient-facing time in clinic is not wasted on tracking down results or reviewing prior medical records. Care gaps can be pre-identified and addressed at that visit. In the setting of juvenile idiopathic arthritis care, this includes having recent lab data and ophthalmology uveitis screening reports available as well as most recent arthritis disease activity scores. Automated PVP reports can be generated from existing data in the PR-COIN registry so not every item has to be manually collected. As pediatric rheumatology sites onboard to PR-COIN, they are encouraged to implement PVP such that it becomes standard in their practices. Effective implementation of PVP saves time for each patient, thereby increasing practice efficiency.

#### Population health management

Population health management (PM) is an approach that aligns with the PR-COIN mission to achieve equitable care and close gaps in care to improve quality measure (QM) performance (19–22). The intent is to leverage clinical information systems (electronic data transfer from EHR systems into the shared registry) to be able to generate reports looking across the entirety of patients in the registry (population), including reports of individual patients who “fail” to pass a measure to prompt action. PM is also critical to avoid loss to follow-up care, particularly of the most vulnerable patients with ongoing active disease. For example, if the goal is to achieve low disease activity or inactive disease, the registry reporting feature can be used to drill down to identify patients with moderate or higher disease activity. A local care coordinator can then conduct outreach based on the reports, e.g., contact patients to schedule visits in case of loss to follow-up and high risk (e.g., moderate disease activity and not seen for >180 days) to be sure treatment is adjusted if the condition is still not under adequate control. PM is an efficient and reliable way to ensure care standards are met across a population. To establish effective PVP, PM at the local hospital level may result in delivery system design changes as part of successful implementation.

#### Shared decision making

A key tenet of the CCM is that disease outcomes will be superior if the patients are invested and engaged in their own healthcare. This led to shared decision making as another network-wide intervention espoused by our LHN (23, 24). PR-COIN developed medication issue cards as a tool for shared decision making to assist patients and families in having discussions with providers that inform selecting their preferred medication regimen to treat their arthritis (25–29). The decision cards focus the discussion on aspects of a medication most important to patients/families such as side effects, frequency of administration, cost, and other factors. In addition to increasing patient engagement, this approach ensures the health care delivery is patient-centered, which is another key element of the CCM. PR-COIN sites have access to these medication issue cards and can utilize them in discussing arthritis medication initiation or changes in therapy.

#### Self-management support

Self-management support (SMS) is the act of empowering or facilitating patients and their family's ability to successfully manage their own medical condition on a day-to-day basis (30, 31). This would incorporate regular assessment of barriers to care and treatment, assistance with finding solutions to problems, and the setting of patient goals with follow up on progress in achieving those goals. PR-COIN launched a network-wide SMS initiative where site members were trained on SMS tools including motivational interviewing, and PR-COIN sites were encouraged to conduct QI work around introducing SMS into practice (32). PR-COIN developed several SMS tools to assist pediatric rheumatology providers, including a SMS change package (33). PR-COIN also adapted The Helping Hands Handbook from Cincinnati Children's Hospital Medical Center. This handbook was created by patients and families with JIA and pediatric rheumatology providers to assist patients and families on their journey navigating life with JIA. This handbook provides information on a wide array of JIA-related topics in limited-literacy and patient-friendly language including education about different aspects of the disease, medications, school accommodations, vaccine considerations and many other components. In addition, PR-COIN team members in conjunction with other researchers created a SMS tool called the barriers assessment tool, which asks the patient/parent to check off different barriers to taking medications including side effects, cost of medication, worry about side effects, forgetting to take medication, and more (33). This tool asks patients to consider these barriers for oral, subcutaneous, and infusion medications as well as barriers to completing occupational and physical therapy. Patients and providers have found the barriers assessment tool helpful at uncovering barriers to care that otherwise might have gone unaddressed. This tool drills down to the root cause of nonadherence to taking medication, which is a problematic aspect in managing chronic diseases such as JIA.

#### Parent and patient engagement

Patient and parent engagement in PR-COIN reflects a commitment to inclusion of all LHN stakeholders in governance, participatory leadership, and in creating a structure for healthcare improvement with quality measures (QMs) that are accountable to patients (34). Parent involvement in co-creation and governance of the network as partners with clinicians and researchers has resulted in a network that has at its core a focus on patient outcomes, and in its heart a focus on meeting the needs of patients and their families. In the goal to be patient-centered, the network has embraced a co-production approach to ensure that the product (delivery of exceptional and equitable health care service and meaningful research) is responsive to the priorities and needs of the patients and families (35). Parents lead and comprise the PR-COIN Engagement Committee, with its associated Parent Working Group (PWG; a parent advisory council) and Patient Advocacy Team (PAT). The PWG/PAT inform and develop patient and family facing educational materials for patient learning and empowerment to foster self-management, reduce barriers to care, and generate tools to enable shared decision making. The parents create public awareness materials (social media, videos) to communicate the work of the network to garner community support and participation.

Parents play a vital role in fostering empathy within the network. Communication of the patient experience is critical for clinicians to become knowledgeable to the impact of health care activities, disease, and its treatment on a personal level. This communication occurs in a manner that is absent or incomplete in the clinic exam room, in which a differential power dynamic, lack of time or other factors may prevent the full disclosure of the scope of disease impact to the clinician. In PR-COIN, there is deliberate intent to remove the hierarchical structure of physician-patient interactions and cultivate a collaborative decision-making setting. Parents and patients are invited to participate as equal partners in all PR-COIN committees bringing the patient perspective to inform and shape network operations and activities and to help set research priorities. Parents present “ignite talks”, create and administer surveys of patient and families to garner broad representative input on topics of network interest, and share and instill the patient voice in network learning sessions and conferences.

The ImproveCareNow LHN has proposed 5 metrics of engagement of patient advisory councils, namely: (1) that there be personal growth for members, (2) internal engagement in community, (3) presence within the LHN, (4) engagement at the local center level, and (5) members contribute to products (36). All of these areas are encouraged in PR-COIN, although engagement at the local center level occurs with variable success. As children move through the system, and invariably transition to adult care so is the need to periodically recruit new parents to work with teams. It can be challenging to meaningfully involve parents into local improvement work, due to their own/family competing interests and job duties during traditional working hours when health care teams meet.

Batalden et al. describe the concept of healthcare as a co-produced service with patients. Likewise, for the LHN model to be effective in design, it requires that it be co-produced by stakeholders, of whom the parents and patients are central (35). In order for PR-COIN to achieve the stated mission that was formulated with parent input, the parents and patients will continue to be involved and represented in the design and measurement of LHN interventions informed in part by their lived experiences. The interventions comprised in the CCM, especially self-management support, underscore the idea of health as a co-produced service. PR-COIN work in the area of shared decision making reflects the steadfast approach of the network towards parents engaged as true partners in care. The PR-COIN registry platform enables parent committee members equal access to shared materials and collaborative files, with protected health information and patient data under separate protection.

While the fundamental drive of LHNs is to reduce unwarranted variation in care to reduce care gaps, increase safety, and promote health equity, the tension with shared decision making and co-production of care is that variation in care may re-enter at the patient level intentionally and according to patient preference (35). This drives home the importance of accurate, health literate and numerate materials to support patient and families to be empowered in informed decision making.

#### Treat to target

“Treat to Target” is an intervention approach that serves to anchor co-production to shared goals of care of the clinician and family (37–39). In this setting, parents select a target for care, classically, “inactive disease” or “low disease activity”. The clinician then works with the family according to guidelines for a Treat to Target approach, which involves systematic assessment of a disease activity measure at regular intervals to allow for adjustment of medication towards reaching the parent/patient goals on disease control or other individual goal (37). A consensus meeting with clinicians and parents highlighted the importance of the patient being able to establish their individual treatment goal, and that it be tracked over time as the treatment plan was adjusted to meet this and other identified goals of care, e.g., disease control, pain control, physical activity, school attendance, etc (38).

### Study of the interventions

PR-COIN regularly reviews QM performance. Select measures are often highlighted during monthly “action period calls”. Furthermore, a deeper dive into the data is done twice a year during the network's “Learning Sessions” when we review measure performance and highlight best practices among sites. Attendees include providers, nurses, other clinical staff, research coordinators, patients/parents, informatic specialists, and registry staff. Additional data review is done at various intervals at a site level, at the coordinating center, during maintenance of certification cycles, and at different committee meetings.

### Measures

PR-COIN has a complete QM set with disease activity outcomes, patient-reported outcomes, process measures, data quality measures, and a balancing measure (1). There are six outcome measures related to disease activity. Our primary outcome measure is patients with oligoarthritis or polyarthritis who have inactive or low disease activity by the 10-joint clinical Juvenile Arthritis Disease Activity Score (cJADAS10). The oligoarthritis group includes patients with the persistent oligoarticular subtype. Polyarthritis includes patients with the International League of Associations for Rheumatology subtypes of extended oligoarticular and polyarticular (both rheumatoid factor negative and positive). The cJADAS10 cut-offs to define inactive or low disease activity for each group were established from the literature, and the cJADAS10 value from the patient's last visit is used (40). Inclusion criteria includes a patient having at least two clinic visits with a clinic visit in the past 450 days. Patients are excluded if they are missing one or more component of the cJADAS10—physician global assessment of disease activity, patient/parent global assessment of overall wellbeing, and active joint count. Another similar measure is the inactive or low disease activity by cJADAS10 by 6 months (after diagnosis). This measure uses the same JIA subtypes and disease activity cut-offs. However, the denominator only focuses on patients recently diagnosed (180–270 days prior), and the measure is reported out quarterly as opposed to monthly for the other measures. A third outcome measure is the mean cJADAS10 score. This measure has similar inclusion and exclusion criteria as our primary measure but assesses cJADAS10 scores from all patients with JIA regardless of subtype.

An additional measure is clinical inactive disease by the American College of Rheumatology (ACR) provisional criteria with the exclusion of inflammatory markers (41). The patient needs to fulfill all five criteria at their last visit to be included in the measure: (1) active joint count of zero, (2) no systemic features (only applicable if a patient has systemic JIA), (3) physician global assessment of disease activity of zero, (4) morning stiffness of 15 min or less, and (5) no current active uveitis. All JIA patients with at least two clinic visits in the past 450 days, with the second visit being at least 180 days after their diagnosis, are eligible for this measure. The patient is excluded if any of the ACR provisional criteria are missing. Additional outcome measures related to disease activity include the mean active joint count and the mean physician global assessment of disease activity score. These measures include all patients with JIA and have similar inclusion and exclusion criteria as our primary measure.

### Analysis

Data are collected at member sites by manual chart review and abstraction and/or electronic data transfer between the EHR and the PR-COIN registry. Site data are pooled to populate collaborative measure data, and this is displayed over time on run charts or control charts. Data span from 2011, when the network was created, to March 2024. Initial center lines are calculated from the initial 20 data points. Special cause on control charts was determined by the presence of two standard control chart rules: (1) shift – 8 or more points in a row above or below the center line and (2) trend – 6 consecutive points increasing or decreasing (42). Furthermore, for run charts, the following standard rules were utilized to determine special cause: (1) shift – 6 or more points in a row above or below the center line and (2) trend – 5 consecutive points increasing or decreasing (42).

### Ethical considerations

The PR-COIN registry protocol was approved by Seattle Children's Institutional Review Board (IRB), which serves as the IRB of record for Seattle Children's Hospital and the following relying participating sites: Stanford University, University of Mississippi, Children's Wisconsin, Northwell Health/Cohen Children's Medical Center, Baylor College of Medicine/Texas Children's Hospital, University of Minnesota, Phoenix Children's Hospital, Nationwide Children's Hospital, Medical University of South Carolina, Hospital for Special Surgery, Hackensack Meridian Health, Cincinnati Children's Hospital Medical Center, Children's Mercy Kansas City, Children's Hospital of Philadelphia, Boston Children's Hospital, and University of Alabama at Birmingham. Due to institutional regulatory policies and local or provincial laws and regulations, the PR-COIN registry protocol was approved by a local IRB for the following participating sites: Levine Children's/Atrium Health (Charlotte, NC, United States), London Health Sciences Centre/Lawson Health Research Institute (London, ON, Canada), McMaster University (Hamilton, ON, Canada), Nemours Orlando (Orlando, FL, United States), Penn State Children's Hospital (Hershey, PA, United States), and The Hospital for Sick Children/SickKids (Toronto, ON, Canada).

## Results

**Figure 2:**
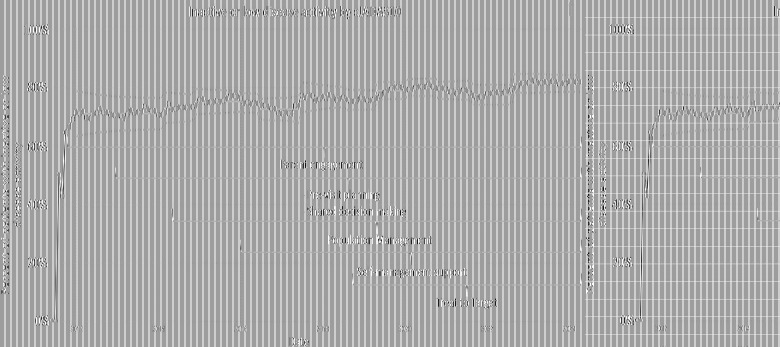
Control chart assessing inactive or low disease activity by cJADAS10. The dots represent our monthly performance. The center line is the mean, and the dashed lines are the upper and lower control limits.

**Figure 3:**
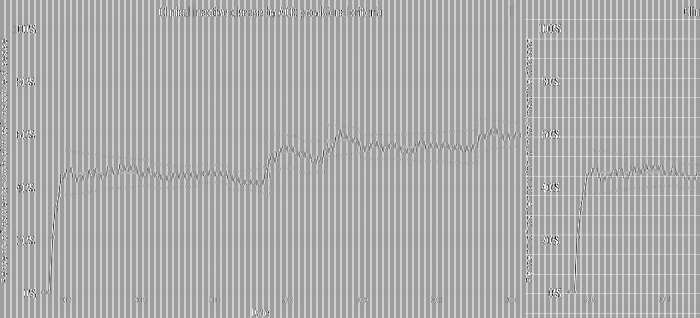
Control chart assessing clinical inactive disease per ACR provisional criteria. The dots represent our monthly performance. The center line is the mean, and the dashed lines are the upper and lower control limits.

**Figure 4:**
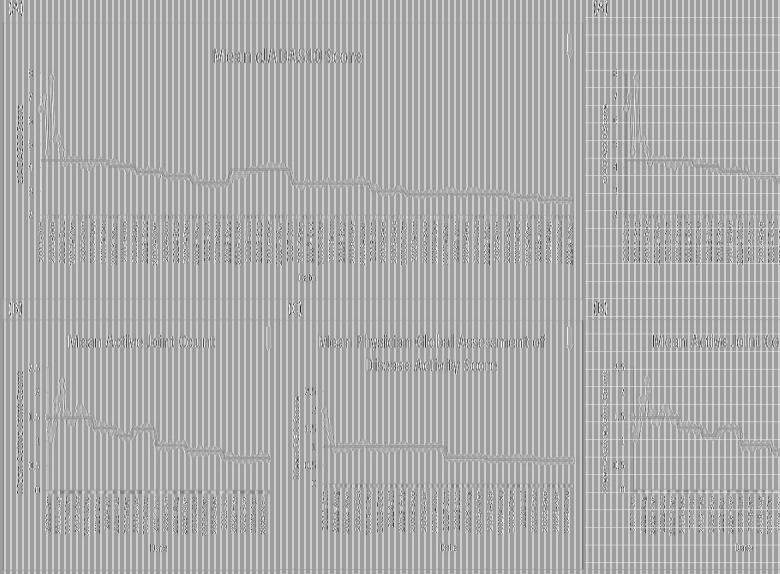
Run charts of the following measures: (**A**) mean disease activity by cJADAS10, (**B**) mean active joint count, and (**C**) mean physician global assessment of disease activity score. The dots represent our monthly performance, and the center line is the median.

Five outcome measures related to disease activity have shown improvement over time. The inactive or low disease activity by cJADAS10 measure (Figure 2) has significantly improved over the last several years with shifts in the data. The initial mean in 2012 and 2013 was 71%, and the current center line is at 81%. The performance for clinical inactive disease by ACR provisional criteria (Figure 3) started at a mean of 46%. After upward shifts, the average collaborative performance is now 60%. The mean disease activity by cJADAS10 measure (Figure 4A) has improved from 4.3 to 2.6 after numerous shifts in the data. In 2011 to mid-2013, the mean active joint count (Figure 4B) was 1.5. This number has significantly decreased over the years with the current center line indicating a mean active joint count of 0.7. Mean physician global assessment of disease activity score (Figure 4C) also significantly improved from 1 to 0.6. The final disease activity outcome measure, inactive or low disease activity by cJADAS10 by six months after diagnosis, has not shown any significant improvement. Quarterly performance from January 2022 to March 2024 has ranged from 30% to 80%.

## Discussion

### Summary

PR-COIN has made significant improvements in the network's disease activity outcome measures for patients with JIA. Furthermore, the network surpassed its goal to have 80% of patients with oligoarticular or polyarticular JIA in an inactive or low disease activity state measured by cJADAS10. Positive substantial change was also noted to the mean cJADAS10 score, the mean active joint count, the mean physician global assessment of disease activity score, and the percent of patients with JIA in clinical inactive disease per ACR provisional criteria.

Overall, PR-COIN's QI initiatives play a vital role in driving continuous improvement in outcome measures for JIA by fostering a culture of learning, collaboration, and innovation within the pediatric rheumatology community. This is the first manuscript highlighting performance on outcome QMs for JIA over time in a quality improvement learning network. PR-COIN's structure and focus on transparency and sharing of best practices has contributed to these improvements in addition to the use of QI methodology both at sites and as a network. PR-COIN's membership across numerous academic pediatric centers throughout the United States and Canada allowed for thousands of patients to be included in these measures, making the results even more meaningful. Numerous network-supported interventions contributed to these improvements including pre-visit planning, shared decision making, self-management support, population management, and a Treat to Target approach to care. Involvement of patients and families in a co-production model has also positively contributed to the network's improvements.

### Interpretation

Direct comparison of our outcome measure performance to other JIA populations in the literature is challenging, and there is a paucity of studies in the literature looking at performance on these validated outcome measures over time. An older Canadian cohort of 16 centers analyzed disease activity outcomes in their combined JIA population; however, direct comparisons to our data is challenging given their different outcome measure definitions and timing of evaluating these outcomes being based off of disease duration (43). This study noted that more than 70% of patients with JIA were in inactive disease within 2 years of diagnosis for all JIA subtypes except rheumatoid factor positive polyarticular JIA patients. Hissink Muller et al. noted that 71% of recent-onset patients with JIA had inactive disease following a 24-month period of providing treat to target-based care (44). Patients with JIA from the Childhood Arthritis and Rheumatology Research Alliance (CARRA) Registry were evaluated at one point in time at least 1 year from diagnosis (45). Forty-six percent of this population had clinical inactive disease by ACR provisional criteria compared to our 60% of patients with JIA. The cJADAS10 measure they reviewed looked at patients with score of 1 or less, which differed from our definition assessing for inactive or low disease activity by established cut-offs by subtype (40). This study's cohort had a median cJADAS10 score of 2 in comparison to our final mean cJADAS10 score of 2.6 (45). Additionally, the authors from the referenced CARRA Registry study noted 51% of patients had a physician global assessment of disease activity score of 0 with median of 0; our last center line of the mean physician global assessment of disease activity score was 0.6.

### Limitations

Our work has some limitations related to data quality. Some sites contribute a relatively small number of patients to each measure given potential local factors including provider engagement, data collection practices, and the time-consuming process of manual data entry. Representativeness of data is lacking in regard to newly diagnosed patients with JIA. This likely contributed to our variability in performance and lack of improvement in our outcome measure of inactive or low disease activity by cJADAS10 by 6 months (after diagnosis). Although 23 sites are participating in the PR-COIN network now, data are actively being entered on a regular basis by 15–17 sites. Transitions between registry platforms have also led to occasional interruptions in data transmission as well.

PR-COIN tracks outcomes for all patients with JIA that can be enrolled in the registry from participating pediatric rheumatology centers. These patients have varying backgrounds and disease severities, which is a strength of this type of analysis, as real-world practice is reflected. This is one reason why data completeness and timeliness are emphasized so that the registry can be representative of all patient populations. There have been teams that have joined and left the network over the past several years that could have influenced QM performance, although they contributed a small number of patients, and the impact is likely minimal. In addition, due to patients aging out of pediatrics, the active patient population changes over time so this is not the same group of patients from year to year.

As highlighted in the Methods section, PR-COIN participates in several network-level interventions or interventional themes. Most of these have overlapping times of initiation/adoption and continued engagement on, which can lead to uncertainty into what interventions directly lead to improvements. Sites also may be working on their own QI projects related to JIA, and the network does not systematically track these individual projects over time. PR-COIN plans to annotate network charts more and encourage sites to track their projects/interventions as well to determine if a change is temporally related. It is possible that changing medication and treatment practices have occurred during the study timeframe that may have partially accounted for a secular trend towards outcome improvement over time.

PR-COIN had a network aim for one of its outcome measures, the inactive or low disease activity by cJADAS10 measure. However, there were no set goals for the other disease activity outcome measures. PR-COIN is actively setting targets now for all of its QMs, and these goals will be reflected on the run charts and control charts going forward. Additional limitations that the network is rectifying include ability to stratify our outcome measure data by numerous variables including race, ethnicity, age, sex assigned at birth, JIA subtype, disease duration, insurance status, and more. Future direction includes ability to consider patient mix when comparing performance across centers. For example, centers with more severe phenotypes (polyarticular, rheumatoid factor positive) unadjusted may show lower rates of disease control compared to other centers. Although the focus of this manuscript was on the disease activity outcome measures, it is important to note that PR-COIN has several patient-reported outcomes that were outside the scope of this manuscript.

## Conclusions

PR-COIN has demonstrated significant improvements in disease activity outcomes for patients with JIA over time. With continued use of QI methodology for both site-specific and collaborative projects, PR-COIN will continue to live out its mission of using QI science to deliver exceptional and equitable health care to children with JIA.

## Data Availability

The datasets presented in this article are not readily available due to legal and IRB requirements. Requests to access the datasets should be directed to PR-COIN@seattlechildrens.org.
